# Dimethyl 3-acetyl-3-(1,3-benzothia­zol-2-yl)penta­nedioate

**DOI:** 10.1107/S1600536808035782

**Published:** 2008-11-08

**Authors:** Yamna Baryala, Moussa Salem, El Mokhtar Essassi, Hans Reuter, Maher Izaaryene

**Affiliations:** a Laboratoire de Chimie Organique et Études Physicochimiques, ENS Rabat, Morocco; b Laboratoire de Chimie Organique Hétérocyclique, Université Mohammed V Rabat, Morocco; c Institute of Chemistry, University of Osnabrück, Barbarastrasse 7, D-49069 Osnabrück, Germany

## Abstract

The title compound, C_16_H_17_NO_5_S, was one of two condensation products from the reaction of 1-(1,3-benzothia­zol-2-yl)propan-2-one with methyl chloro­acetate. The non-H atoms in each of the four substituent groups on the central quaternary C atom are virtually coplanar. The maximum deviations from the least-squares planes are 0.015 (2) and 0.020 (2) Å for the methyl C atoms in the methyl acetate substituents and 0.033 (1) Å for the linked C atom of the benzothia­zole substituent. The S, C and N atoms in the thia­zole ring of the benzothia­zole substituent lie −0.037 (2), 0.046 (2) and −0.028 (2) Å, respectively, from the mean plane defined by the benzene ring atoms.

## Related literature

For general background, see: Palmer *et al.* (1971 ▶); Bénéteau *et al.*, 1999 ▶; El-Sherbeny (2000 ▶); Abayeh *et al.* (1994 ▶); Ivanov & Yuritsyn (1971 ▶); Monsanto (1968 ▶); Lee *et al.* (2001 ▶). For related structures, see: Chen (1994 ▶); Chu *et al.* (2003 ▶).
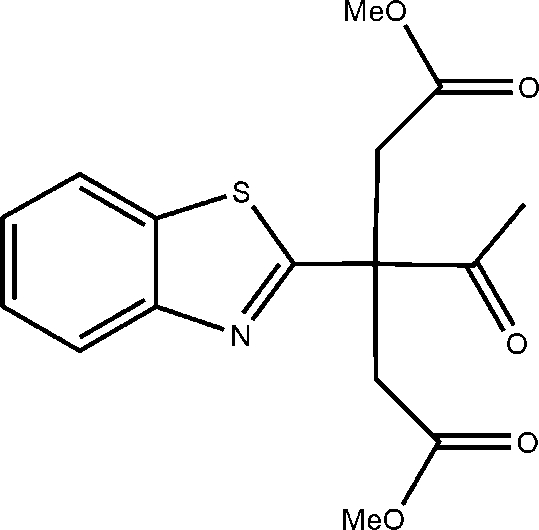


## Experimental

### 

#### Crystal data

C_16_H_17_NO_5_S
*M* *_r_* = 335.37
Monoclinic, 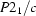

*a* = 14.4075 (3) Å
*b* = 8.8089 (2) Å
*c* = 13.8968 (3) Å
β = 118.011 (1)°
*V* = 1557.10 (6) Å^3^
*Z* = 4
Mo *K*α radiation
μ = 0.23 mm^−1^
*T* = 100 (2) K
0.54 × 0.47 × 0.25 mm

#### Data collection

Bruker APEXII CCD area-detector diffractometer
Absorption correction: multi-scan (*SADABS*; Bruker, 2004 ▶) *T*_min_ = 0.883, *T*_max_ = 0.945
94263 measured reflections
3766 independent reflections
3396 reflections with *I* > 2σ(*I*)
*R*_int_ = 0.030

#### Refinement

*R*[*F* ^2^ > 2σ(*F* ^2^)] = 0.030
*wR*(*F* ^2^) = 0.083
*S* = 1.02
3766 reflections
211 parameters
H-atom parameters constrained
Δρ_max_ = 0.40 e Å^−3^
Δρ_min_ = −0.23 e Å^−3^

### 

Data collection: *SMART* (Bruker, 2004 ▶); cell refinement: *SAINT* (Bruker, 2007 ▶); data reduction: *SAINT*; program(s) used to solve structure: *SHELXS97* (Sheldrick, 2008 ▶); program(s) used to refine structure: *SHELXL97* (Sheldrick, 2008 ▶); molecular graphics: *DIAMOND* (Brandenburg, 1999 ▶); software used to prepare material for publication: *SHELXTL* (Sheldrick, 2008 ▶).

## Supplementary Material

Crystal structure: contains datablocks I, global. DOI: 10.1107/S1600536808035782/fj2155sup1.cif

Structure factors: contains datablocks I. DOI: 10.1107/S1600536808035782/fj2155Isup2.hkl

Additional supplementary materials:  crystallographic information; 3D view; checkCIF report

## Figures and Tables

**Fig. 1.**
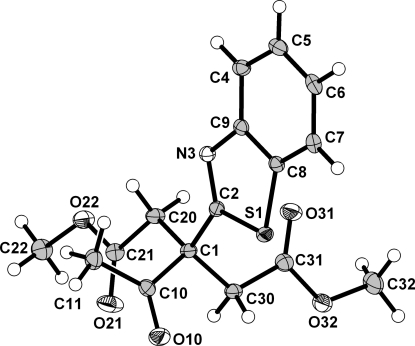
Thermal ellipsoid plot of the title compound with the atomic numbering scheme used. With exception of the hydrogen atoms, which are represented as spheres of arbitrary radius, all other atoms are shown as thermal displacement ellipsoids (oxygen = white, one octant; carbon = grey; nitrogen = white; sulfur = grey, one octant) representing the 50% probability level.

**Fig. 2.**
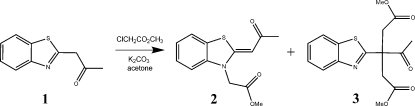
The formation of the title compound.

## supplementary crystallographic information

### Comment

Benzothiazole compounds show interesting biological and pharmacological properties (see, *e.g.*, Palmer *et al.*, 1971; Bénéteau *et al.*, 1999; El-Sherbeny, 2000; Abayeh *et al.*, 1994). Besides some few papers with industrial background (see, *e.g.*, Ivanov & Yuritsyn, 1971; Monsanto, 1968) many papers discuss the synthesis, structure-property relationship and complexation behaviour of these compounds. An important group among the different derivatives of benzothiazole are those substituted at C2 (see, *e.g.*, Lee *et al.*, 2001).

In the following we describe the synthesis and structural characterization of such a kind of benzothiazole derivative. This compound, dimethyl 3-acetyl-3-(1,3-benzothiazol-2-yl)pentanedioate, 3, was synthesized according to reaction scheme 1 through alkylation of 1-(1,3-benzothiazol-2-yl)propan-2-one, 1, with methyl chloroacetate under classical condensation conditions, giving methyl 2-(2-(2-oxopropylidene)1,3-benzothiazol-3(2*H*)-yl)acetate, 2, as second reaction product. The starting material 1 was obtained by condensing 2-aminothiophenol with ethyl acetoacetate in xylene at 160° C for 1 h 30 min.

The structure of the title compound can be best described in relation to the central quaternary carbon atom [C1] surrounded by a benzothiazole moiety, two methyl acetate residues and an acetyl group (Fig. 1). The corresponding bond lengths at C1 of 1.537 (2) Å [C30], 1.538 (2) Å [C20], 1.561 (2) Å [C10] and 1.521 (2) Å are in good agreement with carbon-carbon single bonds, as well as are the angles of 110.9 (1)° - 108.5 (1)° in the range for a tetrahedral coordination. Within the benzothiazole rest bond lengths and angles are very similar to those in comparable compounds like the 2-methyl benzothiazole molecule (Chen, 1994; Chu *et al.*, 2003), which was found as non complexing agent in some crystal structures. Especially the bond angle at the sulfur [88.99 (6)°] and nitrogen [110.5 (1)°] atoms as well as the lengths of the sulfur carbon bonds [1.734 (1)/1.755 (1) A] and the carbon nitrogen double bond between C2 and N3 [129.2 (2) A] are very similar. In relation to the benzenic part [C4 - C9] of the benzothiazole rest which is almost planar [maximal deviation from the least squares plain: -0.0031 (8) Å] the remaining atoms [S1, C2, N3] of the thiazole ring lie -0.037 (2), 0.046 (2) and -0.028 (2) Å above/below this point of reference. This conformation is somewhat different to the 2-methyl benzothiazole molecule where the corresponding values are -0.028 (1), -0.004 (5), 0.017 (4) Å (Chen, 1994) and -0.024 (1), -0.030 (3), -0.017 (3) Å (Chu *et al.*, 2003), respectively, indicating a conformational flexibility of the thiazole moitie. Bond angles and lengths of the two methyl acetate rests as well as those of the acetyl moietie are in the expected ranges [f.e. d(C=O) = 1.201 (2) - 1.206 (2) Å, d(C_carbonyl_—O_methoxy_) = 1.341 (2)/1.332 (2) Å, d(C_methyl_—O) = 1.450 (2)/1.450 (2) Å].

Intermolecular forces are restricted mainly to van der Waals ones. No π-π interactions as well as classical hydrogen bonds are found although there are two short intramolecular contacts between the oxygen atoms of two carbonyl groups and adjacent hydrogen atoms [*d*(O21 - H301) = 2.49 Å, d(O31—H202) = 2.54 Å] and one short intermolecular contact between the sulfur atom of the benzothiazole group and the hydrogen atom of a methylene group [*d*(Sn1—H301) = 2.86 Å] of a neigbhouring molecule.

### Experimental

All solvents and reagents were used as received from Aldrich and Fluka. IR data were recorded using a Bruker VERTEX 70 FTIR spectrometer with ATR device. Wavelengths (ν) are reported in cm^-1. 1^H and ^13^C NMR were obtained at ambient temperature using a Bruker AVANCE 300 A spectrometer. Chemical shifts (δ) are reported in parts per million (p.p.m.) relative to internal standards. Mass spectra were carried out using a LCQ Advantage MAX spectrometer employing Electro Spray Ionization (ESI).

2.84 g (2.61 mmol) methyl chloroacetate were added in one portion to a stirred solution of 100 ml acetone containing 1 g (5.23 mmol) 1-(benzothiazol-2-yl)propan-2-one, 1, and 7.22 g (52.3 mmol) K_2_CO_3_. The reaction mixture was refluxed for 12 h, filtered off and the solvent evaporated. After cooling a pale white precipitate of 2 appeared. These crystals were collected by filtration and recrystallized from ethanol (yield 0.5 g, 36.5%). The filtrate was leaved overnight at room temperature. Thereafter a second crystalline product was obtained which on recrystallization from ethanol gave white single crystals of 3 (yield 0.8 g, 45.6%).

Methyl 2-(2-(2-oxopropylidene)1,3-benzothiazol-3(2*H*)-yl)acetate, 2: mp. 208–210° C; IR (ATR), ν[cm^-1^]): 1608; 1739; ^1^H-NMR (300 MHz; CDCl_3_), δ (p.p.m.): 3.78 (s, 2H), 5.75 (s,1*H*), 6.9–7.6 (m, 4H); ^13^C-NMR (75 MHZ; CDCl_3_), δ (p.p.m.): 29.3; 46,9, 53,1; 90.9; 109.5; 122.7; 123.3; 127.2; 139.4; 160.5; 167.2, 191.5.

Dimethyl 3-acetyl-3-(1,3-benzothiazol-2-yl)pentandioate, 3: mp: 166–168° C; IR (ATR), ν[cm^-1^): 1617; 1718; ^1^H-NMR (30 MHz; CDCl_3_), δ (p.p.m.): 2.24 (s, 3H), 3.54–371 (m, 10H) 7.4–8.0 (m, 4H);^13^C-NMR (75 MHz; CDCl_3_), δ (p.p.m.): 25.7; 38.9; 51.9, 57.5, 121.6, 123.4, 125.5, 126.2, 135.4, 152.4, 152.6 169.8, 171.0, 203.6; MS(ESI): *m/z* 336 [*M+1*]^+^, 639 [*2M+23*]^+^.

A suitable single-crystal of 3 was selected under a polarization microsope and mounted on a 50 µm MicroMesh MiTeGen Micromount^TM ^using FROMBLIN Y perfluoropolyether (LVAC 16/6, Aldrich).

### Crystal data

**Table:**

C_16_H_17_NO_5_S	*F*_000_ = 704
*M**_r_* = 335.37	*D*_x_ = 1.431 Mg m^−^^3^
Monoclinic, *P*2_1_/*c*	Mo *K*α radiation λ = 0.71073 Å
Hall symbol: -P 2ybc	Cell parameters from 9633 reflections
*a* = 14.4075 (3) Å	θ = 2.8–32.7º
*b* = 8.8089 (2) Å	µ = 0.23 mm^−^^1^
*c* = 13.8968 (3) Å	*T* = 100 (2) K
β = 118.011 (1)º	Irregular, colourless
*V* = 1557.10 (6) Å^3^	0.54 × 0.47 × 0.25 mm
*Z* = 4	

### Data collection

**Table:**

Bruker APEXII CCD area-detector diffractometer	3766 independent reflections
Radiation source: fine-focus sealed tube	3396 reflections with *I* > 2σ(*I*)
Monochromator: graphite	*R*_int_ = 0.030
*T* = 100(2) K	θ_max_ = 28.0º
φ and ω scans	θ_min_ = 1.6º
Absorption correction: multi-scan(SADABS; Bruker, 2004)	*h* = −16→19
*T*_min_ = 0.884, *T*_max_ = 0.945	*k* = −11→11
94263 measured reflections	*l* = −18→17

### Refinement

**Table:**

Refinement on *F*^2^	Secondary atom site location: difference Fourier map
Least-squares matrix: full	Hydrogen site location: inferred from neighbouring sites
*R*[*F*^2^ > 2σ(*F*^2^)] = 0.031	H-atom parameters constrained
*wR*(*F*^2^) = 0.083	*w* = 1/[σ^2^(*F*_o_^2^) + (0.0379*P*)^2^ + 0.9909*P*] where *P* = (*F*_o_^2^ + 2*F*_c_^2^)/3
*S* = 1.02	(Δ/σ)_max_ = 0.001
3766 reflections	Δρ_max_ = 0.40 e Å^−^^3^
211 parameters	Δρ_min_ = −0.23 e Å^−^^3^
Primary atom site location: structure-invariant direct methods	Extinction correction: none

### Special details

**Table:**

Geometry. All e.s.d.'s (except the e.s.d. in the dihedral angle between two l.s. planes) are estimated using the full covariance matrix. The cell e.s.d.'s are taken into account individually in the estimation of e.s.d.'s in distances, angles and torsion angles; correlations between e.s.d.'s in cell parameters are only used when they are defined by crystal symmetry. An approximate (isotropic) treatment of cell e.s.d.'s is used for estimating e.s.d.'s involving l.s. planes.
Refinement. Refinement of *F*^2^ against ALL reflections. The weighted *R*-factor *wR* and goodness of fit *S* are based on *F*^2^, conventional *R*-factors *R* are based on *F*, with *F* set to zero for negative *F*^2^. The threshold expression of *F*^2^ > σ(*F*^2^) is used only for calculating *R*-factors(gt) *etc*. and is not relevant to the choice of reflections for refinement. *R*-factors based on *F*^2^ are statistically about twice as large as those based on *F*, and *R*- factors based on ALL data will be even larger.

### Fractional atomic coordinates and isotropic or equivalent isotropic displacement parameters (Å^2^)

**Table:**

	*x*	*y*	*z*	*U*_iso_*/*U*_eq_	
S1	0.07547 (2)	0.86573 (3)	0.43919 (2)	0.01676 (8)	
C2	0.19378 (9)	0.80630 (13)	0.54977 (9)	0.0140 (2)	
N3	0.20635 (8)	0.66132 (11)	0.56438 (8)	0.01443 (19)	
C4	0.11159 (9)	0.42603 (14)	0.47444 (10)	0.0170 (2)	
H4	0.1637	0.3622	0.5273	0.024 (2)*	
C5	0.02392 (10)	0.36495 (14)	0.38745 (10)	0.0190 (2)	
H5	0.0163	0.2578	0.3802	0.024 (2)*	
C6	−0.05382 (10)	0.45859 (15)	0.30984 (10)	0.0193 (2)	
H6	−0.1132	0.4135	0.2508	0.024 (2)*	
C7	−0.04607 (9)	0.61526 (15)	0.31719 (9)	0.0179 (2)	
H7	−0.0991	0.6784	0.2646	0.024 (2)*	
C8	0.04263 (9)	0.67687 (14)	0.40475 (9)	0.0149 (2)	
C9	0.12163 (9)	0.58417 (13)	0.48265 (9)	0.0141 (2)	
C1	0.27346 (9)	0.92387 (13)	0.62176 (9)	0.0145 (2)	
C10	0.22105 (9)	1.00619 (14)	0.68333 (9)	0.0160 (2)	
O10	0.18549 (8)	1.13226 (10)	0.65710 (8)	0.0249 (2)	
C11	0.20998 (10)	0.91849 (15)	0.77027 (10)	0.0219 (3)	
H111	0.2794	0.9052	0.8335	0.0431 (14)*	
H112	0.1792	0.8188	0.7417	0.0431 (14)*	
H113	0.1641	0.9743	0.7922	0.0431 (14)*	
C20	0.37450 (9)	0.84101 (13)	0.70162 (10)	0.0173 (2)	
H201	0.3553	0.7532	0.7331	0.0431 (14)*	
H202	0.4102	0.8014	0.6608	0.0431 (14)*	
C21	0.45015 (9)	0.94072 (14)	0.79307 (10)	0.0170 (2)	
O21	0.43873 (8)	1.07423 (11)	0.80278 (8)	0.0304 (2)	
O22	0.53173 (7)	0.86031 (10)	0.86465 (8)	0.0229 (2)	
C22	0.60859 (10)	0.94308 (15)	0.95845 (11)	0.0232 (3)	
H221	0.6652	0.8742	1.0055	0.0431 (14)*	
H222	0.5747	0.9852	0.9993	0.0431 (14)*	
H223	0.6379	1.0258	0.9339	0.0431 (14)*	
C30	0.29294 (10)	1.04297 (13)	0.55238 (10)	0.0168 (2)	
H301	0.2280	1.1038	0.5129	0.0431 (14)*	
H302	0.3489	1.1126	0.6021	0.0431 (14)*	
C31	0.32428 (9)	0.98185 (14)	0.47030 (10)	0.0177 (2)	
O31	0.34329 (8)	0.85225 (11)	0.45908 (8)	0.0272 (2)	
O32	0.32806 (9)	1.09691 (11)	0.40907 (8)	0.0281 (2)	
C32	0.36038 (13)	1.05932 (17)	0.32765 (12)	0.0309 (3)	
H321	0.3587	1.1509	0.2869	0.0431 (14)*	
H322	0.3123	0.9833	0.2774	0.0431 (14)*	
H323	0.4320	1.0183	0.3636	0.0431 (14)*	

### Atomic displacement parameters (Å^2^)

**Table:**

	*U*^11^	*U*^22^	*U*^33^	*U*^12^	*U*^13^	*U*^23^
S1	0.01835 (15)	0.01332 (14)	0.01468 (14)	0.00202 (10)	0.00450 (11)	0.00100 (10)
C2	0.0143 (5)	0.0144 (5)	0.0137 (5)	0.0014 (4)	0.0071 (4)	0.0008 (4)
N3	0.0153 (4)	0.0135 (5)	0.0152 (5)	−0.0003 (4)	0.0078 (4)	−0.0001 (4)
C4	0.0197 (6)	0.0144 (5)	0.0205 (6)	0.0007 (4)	0.0124 (5)	0.0000 (4)
C5	0.0231 (6)	0.0159 (6)	0.0229 (6)	−0.0043 (5)	0.0150 (5)	−0.0047 (5)
C6	0.0201 (6)	0.0236 (6)	0.0165 (5)	−0.0058 (5)	0.0105 (5)	−0.0067 (5)
C7	0.0184 (5)	0.0222 (6)	0.0134 (5)	0.0001 (5)	0.0077 (5)	−0.0014 (4)
C8	0.0178 (5)	0.0144 (5)	0.0153 (5)	0.0001 (4)	0.0100 (5)	−0.0011 (4)
C9	0.0155 (5)	0.0150 (5)	0.0146 (5)	−0.0009 (4)	0.0094 (4)	−0.0010 (4)
C1	0.0161 (5)	0.0113 (5)	0.0160 (5)	0.0002 (4)	0.0074 (4)	0.0002 (4)
C10	0.0153 (5)	0.0164 (6)	0.0149 (5)	−0.0015 (4)	0.0059 (4)	−0.0022 (4)
O10	0.0319 (5)	0.0192 (5)	0.0284 (5)	0.0088 (4)	0.0181 (4)	0.0030 (4)
C11	0.0260 (6)	0.0235 (6)	0.0189 (6)	−0.0034 (5)	0.0127 (5)	−0.0007 (5)
C20	0.0157 (5)	0.0131 (5)	0.0193 (6)	0.0001 (4)	0.0050 (5)	−0.0006 (4)
C21	0.0146 (5)	0.0172 (6)	0.0189 (6)	−0.0013 (4)	0.0077 (5)	−0.0003 (4)
O21	0.0262 (5)	0.0173 (5)	0.0323 (5)	0.0016 (4)	0.0011 (4)	−0.0066 (4)
O22	0.0195 (4)	0.0179 (4)	0.0214 (4)	−0.0004 (3)	0.0013 (4)	−0.0009 (4)
C22	0.0160 (5)	0.0240 (6)	0.0220 (6)	−0.0038 (5)	0.0026 (5)	−0.0027 (5)
C30	0.0211 (6)	0.0121 (5)	0.0191 (6)	−0.0011 (4)	0.0110 (5)	−0.0004 (4)
C31	0.0172 (5)	0.0173 (6)	0.0187 (6)	−0.0036 (4)	0.0086 (5)	−0.0019 (5)
O31	0.0409 (6)	0.0178 (5)	0.0324 (5)	0.0027 (4)	0.0250 (5)	−0.0007 (4)
O32	0.0501 (6)	0.0179 (5)	0.0291 (5)	−0.0045 (4)	0.0290 (5)	−0.0020 (4)
C32	0.0486 (9)	0.0270 (7)	0.0308 (7)	−0.0086 (6)	0.0300 (7)	−0.0046 (6)

### Geometric parameters (Å, °)

**Table:**

S1—C8	1.7343 (12)	C11—H112	0.9800
S1—C2	1.7552 (12)	C11—H113	0.9800
C2—N3	1.2924 (15)	C20—C21	1.5078 (16)
C2—C1	1.5207 (16)	C20—H201	0.9900
N3—C9	1.3926 (15)	C20—H202	0.9900
C4—C5	1.3827 (18)	C21—O21	1.2038 (16)
C4—C9	1.3997 (16)	C21—O22	1.3320 (15)
C4—H4	0.9500	O22—C22	1.4496 (15)
C5—C6	1.4012 (18)	C22—H221	0.9800
C5—H5	0.9500	C22—H222	0.9800
C6—C7	1.3845 (18)	C22—H223	0.9800
C6—H6	0.9500	C30—C31	1.5095 (16)
C7—C8	1.3957 (17)	C30—H301	0.9900
C7—H7	0.9500	C30—H302	0.9900
C8—C9	1.4060 (16)	C31—O31	1.2012 (15)
C1—C30	1.5373 (16)	C31—O32	1.3409 (15)
C1—C20	1.5383 (16)	O32—C32	1.4497 (16)
C1—C10	1.5605 (16)	C32—H321	0.9800
C10—O10	1.2055 (15)	C32—H322	0.9800
C10—C11	1.5043 (17)	C32—H323	0.9800
C11—H111	0.9800		
			
C8—S1—C2	88.99 (6)	C10—C11—H113	109.5
N3—C2—C1	124.25 (10)	H111—C11—H113	109.5
N3—C2—S1	116.04 (9)	H112—C11—H113	109.5
C1—C2—S1	119.70 (8)	C21—C20—C1	113.34 (10)
C2—N3—C9	110.46 (10)	C21—C20—H201	108.9
C5—C4—C9	118.40 (11)	C1—C20—H201	108.9
C5—C4—H4	120.8	C21—C20—H202	108.9
C9—C4—H4	120.8	C1—C20—H202	108.9
C4—C5—C6	121.04 (11)	H201—C20—H202	107.7
C4—C5—H5	119.5	O21—C21—O22	123.70 (11)
C6—C5—H5	119.5	O21—C21—C20	125.51 (11)
C7—C6—C5	121.49 (11)	O22—C21—C20	110.77 (10)
C7—C6—H6	119.3	C21—O22—C22	115.95 (10)
C5—C6—H6	119.3	O22—C22—H221	109.5
C6—C7—C8	117.45 (11)	O22—C22—H222	109.5
C6—C7—H7	121.3	H221—C22—H222	109.5
C8—C7—H7	121.3	O22—C22—H223	109.5
C7—C8—C9	121.61 (11)	H221—C22—H223	109.5
C7—C8—S1	129.28 (10)	H222—C22—H223	109.5
C9—C8—S1	109.11 (9)	C31—C30—C1	115.97 (10)
N3—C9—C4	124.73 (11)	C31—C30—H301	108.3
N3—C9—C8	115.28 (10)	C1—C30—H301	108.3
C4—C9—C8	120.00 (11)	C31—C30—H302	108.3
C2—C1—C30	110.85 (9)	C1—C30—H302	108.3
C2—C1—C20	108.53 (9)	H301—C30—H302	107.4
C30—C1—C20	112.75 (10)	O31—C31—O32	123.80 (11)
C2—C1—C10	105.49 (9)	O31—C31—C30	127.20 (11)
C30—C1—C10	107.74 (9)	O32—C31—C30	109.00 (10)
C20—C1—C10	111.26 (9)	C31—O32—C32	116.51 (11)
O10—C10—C11	121.71 (11)	O32—C32—H321	109.5
O10—C10—C1	120.57 (11)	O32—C32—H322	109.5
C11—C10—C1	117.58 (10)	H321—C32—H322	109.5
C10—C11—H111	109.5	O32—C32—H323	109.5
C10—C11—H112	109.5	H321—C32—H323	109.5
H111—C11—H112	109.5	H322—C32—H323	109.5
			
C8—S1—C2—N3	−3.31 (9)	N3—C2—C1—C10	−111.43 (12)
C8—S1—C2—C1	177.11 (9)	S1—C2—C1—C10	68.12 (11)
C1—C2—N3—C9	−177.68 (10)	C2—C1—C10—O10	−104.69 (13)
S1—C2—N3—C9	2.75 (12)	C30—C1—C10—O10	13.77 (15)
C9—C4—C5—C6	−0.72 (17)	C20—C1—C10—O10	137.83 (12)
C4—C5—C6—C7	−0.08 (18)	C2—C1—C10—C11	71.08 (12)
C5—C6—C7—C8	0.34 (17)	C30—C1—C10—C11	−170.47 (10)
C6—C7—C8—C9	0.21 (17)	C20—C1—C10—C11	−46.41 (14)
C6—C7—C8—S1	−178.77 (9)	C2—C1—C20—C21	−167.17 (9)
C2—S1—C8—C7	−178.22 (11)	C30—C1—C20—C21	69.63 (13)
C2—S1—C8—C9	2.70 (8)	C10—C1—C20—C21	−51.54 (13)
C2—N3—C9—C4	179.46 (11)	C1—C20—C21—O21	−3.89 (18)
C2—N3—C9—C8	−0.50 (14)	C1—C20—C21—O22	174.63 (10)
C5—C4—C9—N3	−178.70 (10)	O21—C21—O22—C22	0.40 (18)
C5—C4—C9—C8	1.25 (17)	C20—C21—O22—C22	−178.16 (10)
C7—C8—C9—N3	178.94 (10)	C2—C1—C30—C31	−53.93 (13)
S1—C8—C9—N3	−1.90 (12)	C20—C1—C30—C31	67.97 (13)
C7—C8—C9—C4	−1.02 (17)	C10—C1—C30—C31	−168.88 (10)
S1—C8—C9—C4	178.15 (9)	C1—C30—C31—O31	−6.12 (19)
N3—C2—C1—C30	132.23 (12)	C1—C30—C31—O32	173.84 (10)
S1—C2—C1—C30	−48.22 (12)	O31—C31—O32—C32	−1.96 (19)
N3—C2—C1—C20	7.89 (15)	C30—C31—O32—C32	178.08 (11)
S1—C2—C1—C20	−172.56 (8)		
